# Supplementary material for: Impact of being an adolescent mother on subsequent maternal health, parenting, and child development in Kenyan low-income and high adversity informal settlement context
Source: PLoS One. 2021 Apr 1;16(4):e0248836. doi: 10.1371/journal.pone.0248836 (PMC8016237; doi:10.1371/journal.pone.0248836)
Supplement: Attachment — Submitted filename: letter of review.docx [file pone.0248836.s002.docx]

Thank you for the opportunity to review this very interesting review study. The manuscript was well-organized. There are a number of aspects which require revision to make this paper suitable for publication

1. Sampling does not clear, what the sampling method and the sample size calculation
2. why the sample size of the two groups is different?
3. Inclusion criteria should be clear, the participants were mother who over 18 years or younger when they giving birth? Please clearly define of adolescent mother
4. Give more detail related study setting located
5. Explain the aims at the beginning of discussion part
6. The discussion of findings is good, but rather need strengthen discussion related result especially for child internalizing problems and child physical health
7. Discussion related Adolescent mothers have poor maternal mental health as adults only focus on results, need more strengthen
