# Supplementary material for: Impact of being an adolescent mother on subsequent maternal health, parenting, and child development in Kenyan low-income and high adversity informal settlement context
Source: PLoS One. 2021 Apr 1;16(4):e0248836. doi: 10.1371/journal.pone.0248836 (PMC8016237; doi:10.1371/journal.pone.0248836)
Supplement: Attachment — Submitted filename: PLOS ONE reviewers comments_YH_Ed_Nov30.docx [file pone.0248836.s003.docx]

**PONE-D-20-09731 -Impact of being an Adolescent Mother on Subsequent Maternal Health, Parenting, and Child Development in Kenyan low-income and high adversity informal settlement context -PLOS ONE**

| **Reviewer** | **Comments** | **Response** |
| --- | --- | --- |
| Editor’s comments | In addition, as editor I would raise the issues that the clarity of the study's objectives would be enhanced if outcomes for mothers and children were described separately.  Adolescent pregnancy is the exposure variable, but it is treated as a maternal outcome variable in the Measures section.  The Sample is not representative, respondents were not chosen on a probability basis.   The SEM models claims a mediating effect on child physical health, but since there was no main effect, this does not meet criteria (Barron and Kenney).  Figure 1 a and b are titled "Paternal" but I believe this is a typo, please correct.  Finally, the manuscript must be edited for scientific English grammar; there are problems with subject-verb agreement, sentence structure, articles (lack of), etc. | Dear editor, thanks for these comments and we have corrected the typos. We have separated the maternal and child outcomes and you are right that this wasn’t a sample selected on probability basis but one where we felt post-data collection that history of their motherhood and parental journey had an important story to be learnt from. We have worked on the language and our apologies for the oversight with grammatical errors.  For the SEM analysis, we now clarify that we using Stress-Process/SDH path model. We test whether adolescent pregnancy is associated with poor subsequent adulthood health and parenting, which further influences child health/development outcomes |
| Reviewer 1  General questions and comments | while the methodology is generally sound – the study rationale, theoretical framing, and interpretation and discussion of results are weak. Below please find general comments and specific questions | Dear reviewer 1, thanks for your feedback and we have now strengthened the discussion and clarify our theoretical framework as explained above. |
|  | The conceptual framework is unclear. I do not see Figure 1, as referenced by the authors as illustration of how they conceptualize SDOH in this context. Further, structural determinants (e.g. education) appear to be used as control variables, rather than covariates of substantive interest. This is not consistent with a SDOH logic, which would center the measurement and effects of macro-level features of the local society – which appears to be quite unique (see below). I also note that the authors’ later mention in the discussion “All three frameworks, family stress model, social determinants of health and adverse child experiences, that we have discussed here provide critical rejoinders….” I do not see any reference to the family stress model or adverse child experiences, as they relate to the study | We have corrected this and maintained two here – ACE and SDH as a reference point.  We now also clarify the study was based on an “Integrated Stress and SDH Process framework for health disparities”, and study 3 objectives (see revised background).  We thank you for the opportunity to rethink our framework. |
|  | In the background section, the authors conflate the literature on early/adolescent motherhood, unintended motherhood and single motherhood – yet these are distinct, although sometimes overlapping, phenomena. I encourage the authors to be more specific and explicit in their summary of the existing literature, particularly from LMICs. In particular, I would want to know how this manuscript advances our knowledge of the long-term effects of adolescent motherhood in the context of Kenya/East Africa | This is an important observation and the mental health outcomes emanating from all three- unintended motherhood, early motherhood and single motherhood are unclear. We now note that existing literature did not usually distinguish short- and long-term impacts adolescent pregnancy. Our study address this gap by examining whether adolescent motherhood is associated with cascade of health problems as mother aged (or related to different patterns of health disparities as mother aged) through examining health disparities patterns by mothers’ age groups (See Objective section). We also clarify the contribution of this study (in 3 ways) in the Discussion section. |
|  | In many places, the discussion of results is extremely general and not specific to the study itself. For example, the authors suggest that the ultimate focus needs to be on sustainably poverty eradication measures. This statement is not clearly supported by the study design or results. What measures of poverty are included? The second section on WHO theoretical frameworks reads as a generic summary of existing frameworks, rather than a specific discussion of how the study results advance discussion of development priorities. The authors mention a Figure 3, which I do not see included in the manuscript | We apologize for not being clear. We have corrected the mistake and clarify our theoretical framework that guide the measurement and research questions. |
|  | Finally, I am curious about the study site. This appears to be a unique setting. Indeed, the authors explicitly mention that this is a “low income, high adversity informal settlement context” in the manuscript title, and of course, they reference structural and social conditions that influence health in their discussion of the SDOH framework. Yet, there is inadequate explanation of the context, and no apparent measurement of local social or structural conditions. For example, if this was an informal settlement, why were no measures of migration included? The authors mention it is a high-adversity context. Why are no measures of violence or adversity included? What is relevant about the study site? I would want to know why was the study conducted here, how did measures account for the local setting, and how might the results of this study be affected by the socio-cultural conditions of place | We have worked on two informal settlements and the sites have been described on the Methods section. The context we provided is based on qualitative information from the communities. We do not have quantifiable indicators/measures for community violence, social and structural conditions (as the reviewer requested) given these community indicators are not tracked by local government.  Going forward the observations of the reviewers are very helpful and we will be considering those while designing the study.  The study was part of a survey we carried out in two primary health care facilities of Nairobi which neighbour large informal settlements. We wanted to look at parenting, food insecurity and child mental health outcomes for ages 5-19 years. |
|  | The authors’ note that the Kenya government recorded 350,000 adolescent pregnancies between 2016-2017 and that this is a rise from previous estimates. Yet the previous estimate was published in 2017? How do we know this is a substantive increase, and not just general fluctuation? Is there available trend data that the authors could use to make this point better? | We have now amended and added KDHS 2014 data and some findings from 2019 too. We thank you for pointing that out. |
|  | Among women who were adolescent mothers, were the children sampled the children who were born before the mother was age 18? | Yes |
|  | The authors describe this as a female caregiver sample. Please provide greater detail. Are they caregivers because they were bringing children to the health clinic? | Yes and they were primary caregivers |
|  | Were interviews conducted by women? | By female clinical psychologists |
|  | Please include measurement details for all covariates. For example, how was food insecurity measured? | We now add the description for the covariate measures in the Measures section. |
|  | Use of causal language should be avoided, given the cross-sectional data. For example, the regression models do not provide evidence of impact of adolescent motherhood on maternal education, employment and food security. They provide evidence for association. The pathway could be bidirectional | This is well noted. We have modified our language accordingly. |
|  | The study appears to use measures of child’s biological sex, not gender. I encourage the authors to make edits where appropriate | We appreciate the suggestion, and have make edits where appropriate. |
|  | Why are figures of ANCOVA analyses presented only for maternal health, and not also for parenting practices and child health? Further, the results also seem to indicate need for mental health interventions with older women, not only preventative work with younger women | Yes that is a point we have now incorporated. But we were making a case for early intervention targeting adolescents. but we do agree it is across the lifespan |
|  | Why is food insecurity not included as a control variable for regression and SEM models? | Thank you for the suggestion. We now include food insecurity in the SEM. |
|  | In many places, there is a disconnect between sequential sentences, which inhibits flow of reading and comprehension. For example, “Furthermore, adolescent pregnancy also has severe medical and obstetric complications. One study from UK [sic] found that the early age at first birth is associated with poorer mental health…” In this example, I would be interested to learn also about the medical and obstetric complications and be pointed toward key references | We have reorganized the paper to improve the flow and readability of the paper |
|  | What do you mean by references to conflict marriage? Abusive relationships? What is the prevalence of IPV in this sample? If it was measured, I would strongly recommend including IPV in the analysis, given its potential role as a confounding variable | Sadly we did not measure this but we do know of this from literature |
|  | The manuscript requires a comprehensive copy-edit. There are extensive grammatical and spelling errors throughout the document | We have done extensive edit to improve the manuscript. |
| Reviewer 2 | Sampling does not clear, what the sampling method and the sample size calculation | We now describe study design and sampling representation in the Participant section. |
|  | why the sample size of the two groups is different? | We clarify data are derived from a cross-sectional study (not a cohort design, with equal number of expose and un-expose sample). Therefore, group size is different. |
|  | Inclusion criteria should be clear, the participants were mother who over 18 years or younger when they giving birth? Please clearly define of adolescent mother | We have clarified that now and thank you for your feedback. |
|  | Give more detail related study setting located | We have added that now. |
|  | Explain the aims at the beginning of discussion part | We now add the Aims/purpose of the study in the beginning of the Discussion section |
|  | The discussion of findings is good, but rather need strengthen discussion related result especially for child internalizing problems and child physical health | We have revised the discussion and incorporated the suggestion |
|  | Discussion related Adolescent mothers have poor maternal mental health as adults only focus on results, need more strengthen | We have incorporated the suggestion and add theoretical explanation. |
